# Cataract significantly influences quantitative measurements on swept-source optical coherence tomography angiography imaging

**DOI:** 10.1371/journal.pone.0204501

**Published:** 2018-10-02

**Authors:** Siqing Yu, Beatrice E. Frueh, Dagmar Steinmair, Andreas Ebneter, Sebastian Wolf, Martin S. Zinkernagel, Marion R. Munk

**Affiliations:** Department of Ophthalmology and Department of Clinical Research, Inselspital, Bern University Hospital, University of Bern, Bern, Switzerland; Massachusetts Eye & Ear Infirmary, Harvard Medical School, UNITED STATES

## Abstract

**Purpose:**

To analyze retinal blood flow before and after cataract surgery using swept-source optical coherence tomography angiography (SS-OCTA).

**Methods:**

Prospective observational study. Consecutive patients were recruited and scanned using SS-OCTA before and after cataract surgery. Laser flare photometry were performed post-surgery. Perfusion and vessel density of superficial (SCP) and deep capillary plexuses (DCP) of the 3 × 3 mm images as well as foveal avascular zone (FAZ) measurements were assessed. Vessel continuity, vessel visibility and presence of artefacts were evaluated by two blinded graders using a predefined grading protocol.

**Results:**

Thirteen eyes of 12 patients met the inclusion criteria. There was a significant increase of perfusion and vessel densities in both the SCP and the DCP after cataract surgery within the 3 × 3 mm images. Significantly better distinguishability of FAZ border was observed postoperatively in both SCP and DCP, however, FAZ area and perimeter measurements did not significantly change after cataract surgery. Mean number of motion artifacts in SCP and DCP numerically decreased by 37% (P = .089) and 42% (P = .080).

**Conclusions:**

Lens opacities have a significant influence on retinal blood flow measurements in SS-OCTA and should be considered in quantitative vessel analysis. Inflammation may also impact the assessment of density parameters. FAZ measurements seems to be the most robust parameters in terms of media opacity.

## Introduction

Optical coherence tomography angiography (OCTA) is the latest imaging modality that can be used to image the microvasculature of retina and choroid without dye injection. Instead of staining vasculature as fluorescein angiography (FA), OCTA is based on the concept of “motion contrast”. It visualizes blood flow by detecting dynamic structures among other static tissues such as the neurosensory retina. Thus, with its features of non-invasiveness, repeatability, OCTA rapidly gained widespread usage in the investigation, evaluation and monitoring of retinal and choroidal vascular diseases.[1–3]

Spectral-domain (SD) and swept-source optical coherence tomography (SS-OCT) are the most common OCT technologies. SD-OCT instruments use a broadband near-infrared superluminescent diode as a light source, with a wavelength of approximately 870 nm. SS-OCT instruments use a tunable swept laser, with a longer wavelength of approximate 1050 nm. Longer wavelength results in better tissue penetration, as well as better detection of signals from the deeper layers. Accordingly, SS-OCT systems do not only overcome the barrier of the RPE, but also offer improved reliability to ocular opacities such as cataract.[4] Additionally, scanning speed of SS-OCTA is twice as fast, which allows for denser scan patterns and larger scan areas in a given time period.

However, despite fast imaging acquisition, image artifacts are still unavoidable in SS-OCTA. The majority of artifacts is motion-related, such as eye movements and blink, which induce white lines, black bands, vessel-displacement or -doubling and out-of-window artifacts. Other artefacts such as projection artefacts or masking artifacts are found as well.[5] At least one of these artifacts was observed in 89% examinations in previous studies. Compared to young healthy controls, image artifacts are more common in patients with ocular pathology and poor visual acuity.[6,7] Nevertheless, respective artefacts were believed not to interfere with qualitative image interpretation.[8]

Many patients with retinal pathologies, which are nowadays evaluated with OCTA are of older age and have already developed significant cataract. However, so far no study is existing, which accurately evaluated, whether respective opacity can interfere with evaluation and might bias assessed parameters.

In this prospective study, we analyzed retinal blood flow of cataract patients before and after cataract surgeries using SS-OCTA to investigate its accuracy and reliability.

## Methods

### Patients and setting

In this prospective observational study, patients were consecutively recruited from the Department of Ophthalmology, University Hospital Bern, Switzerland. Collection and analysis of SS-OCTA images were approved by the ethics committee of the University of Bern, Switzerland, and was conducted in compliance with the tenets of the Declaration of Helsinki. Informed consents were obtained from all participants.

22 consecutive patients scheduled for phacoemulsification, and intraocular lens implantation were enrolled. A thorough ophthalmic exam was completed on every patient before and after surgery, including Early Treatment Diabetic Retinopathy Study (ETDRS) best-corrected visual acuity (BCVA) assessing with Snellen chart, slit-lamp Goldman applanation tonometry, pupil dilation, color fundus photograph and SD-OCT (Spectralis, Heidelberg Engineering, Germany). Cataract severity was assessed using the Lens Opacities Classification system III (LOCS scale).[9] Patients diagnosed with glaucoma or any retinopathies that might result in abnormal microvasculature (e.g., age-related macular degeneration, diabetic retinopathy, retinal vascular disorder, etc.) and previously treated by laser or photodynamic therapy were also excluded. Preoperative SS-OCTA exam was performed on the day before the operation. To be included in the analysis, scans had to show signal strength scores greater than 6 to allow quantitative assessment at all. If eyes did not meet this criterion, no follow up SS-OCTA scans were performed and patients were only assessed for cataract severity and BCVA to allow comparison. Due to media opacity and unsteady fixation, 10 patients had low OCTA signal strength scores of less than 6 and did not meet image quality criterion and were therefore excluded from image analysis. Postoperative SS-OCTA images were therefore obtained in the remaining 13 eyes of 12 patients on the follow-up visit about 1 week after the surgery. Laser flare photometry (FM-600; Kowa Company, Ltd., Nagoya, Japan) was performed on the follow-up visit to quantify inflammatory response.

### Image acquisition

3 mm × 3 mm raster scans centered on the fovea were obtained by a 100-kHz SS-OCTA instrument (PLEX Elite 9000; Carl Zeiss Meditec, Inc, Dublin, CA) with a central wavelength of 1060 nm, an axial resolution (optical) of 6.3 μm, and a lateral resolution of 20 μm. The scanning rate is 100 000 A-scans per second. It consists of 300 A scans per B scan (with 10 μm spacing between adjacent scans), 4 B-scan repetitions per location, and 300 B-scan positions in the raster. The en-face images of the superficial capillary plexus (SCP) were captured using the customized segmentation between an inner boundary at the inner limiting membrane (ILM) and an outer boundary at the inner plexiform layer (IPL), while the deep capillary plexus (DCP) were visualized between the IPL and the outer plexiform layer (OPL).

All scans were automated analyzed using the macular density algorithm (v0.6.1) developed by ZEISS Algorithm Development-Macular Density and fovea avascular zone (FAZ) Metrics. Perfusion density and vessel density of SCP and DCP were measured. Perfusion density is defined as the total area of perfused vasculature per unit area in a region of measurement. The unitless results range from 0 (no perfusion) to 1 (fully perfused). Vessel density refers to the total length of perfused vasculature per unit area in a region of measurement in units of inverse millimeters. DCP parameters were calculated after removing the projection artifacts from the SCP. Perfusion and vessel density were analyzed in three respective areas: inner 3 mm ETDRS ring (henceforth named inner ring), inner 3 mm including the central 1 mm and the 3 mm ETDRS ring (henceforth named inner 3 mm) and the whole 3 mm × 3 mm scan area (henceforth named 3 × 3 mm). FAZ boundary was outlined along the innermost capillaries on the SCP slab and was used to measure the perimeters of FAZ. FAZ measurements were reported in area, perimeter and circularity index. FAZ circularity index was defined as the ratio of the perimeter of the FAZ and the perimeter of a circle with equal area, and is supposed to indicate vascular dropout, for it decreases as the shape becomes less round or less smooth.[10]

### Grading protocol

The scans were checked for segmentation errors and subsequently, the en-face OCTA images of the SCP and DCP were exported and masked. Then they were qualitatively and quantitatively evaluated for continuity of the vessels and presence of artefacts by 2 independent, experienced retinal imaging experts (S.Y. and M.R.M) according to a pre-specified grading protocol.[11] Briefly, the grading scale included 5 parameters: motion and image artefacts (1 = no artefacts, 0 = 1–4 artefacts, -1 = ≥ 5 artefacts or severe artefacts making reasonable evaluation of the microvasculature impossible), distinguishability of FAZ border (1 = FAZ border well distinguishable, 0 = middle, -1 = FAZ border barely/not distinguishable), and vessel continuity and discriminability of large and small vessels (1 = vessel continuity clearly preserved, 0 = middle, -1 = vessel continuity not preserved). Vessel continuity and discriminability of large vessels were only graded in SCP images.

As described in previous studies, motion artifacts were considered as present when there were doubling or displacement of retinal vessels, or characteristic white-line with corresponding discontinuity on the en-face image in B-scan direction, or continuous dark bands of varying width due to blink. Image artefacts were defined as any anomaly in addition to motion artefacts, such as segmentation artefacts, which leads to deviation of the slab; projection artefacts, which were from more superficial vasculature projecting on deeper structures; masking artefacts, which were low signal region blocked by the more anterior dense; loss of focus, ect.[5–7,12–15] Additionally, the number of clearly identifiable bifurcations in the SCP en-face images was counted to evaluate the vessel visibility. The counting procedure was as follows: a main, large vessel branch at 12 o`clock was chosen, and the number of identifiable, subsequent bifurcations towards the terminal capillary end were counted on the respective branch.[11]

After concordance analyses and the evaluation of the inter-grader reliability, a consensus grading was performed, including an individual score for each feature of each image.

### Statistical analyses

SPSS (IBM, SPSS statistics, Version 21; SPSS Inc, Chicago, IL) was used for statistical analysis. Inter-grader reliability was quantified by Cohen's kappa. Numeric data were presented as mean ± standard deviation and analyzed by Student’s t-test. For categorical variables, such as the individual scores (ranging from -1/0/+1) in the consensus grading, Pearson’s χ^2^ test were used. Correlations between laser flare and density parameters were analyzed by Spearman's correlation. Individual scores of each feature were summed up and normalized as a final score ranging from -100 to +100. P value of < .05 was considered statistically significant.

## Results

### Characteristics of the study population

Of the 22 enrolled patients, 10 patients were excluded due to low quality scans. Adequate preoperative quality SS-OCTA images were obtained in 13 eyes of 12 patients. Characteristics of these 13 eyes and the excluded eyes are presented in Table 1. The mean age of these 12 patients was 71.2 ± 10.7 years (range 50–86 years). Mean BCVA before surgery was .500 ± .148 (range .3-.8). Median LOCS scale was N4, C3 and P4. The mean OCTA signal strength score reported by the OCTA device before surgery was 6.85 ± .555 (range 6–8). The included study patients were younger, had significantly better BCVA, LOCS scale and OCTA signal strength compared to the excluded ones.

**Table 1:** Comparison of characteristics in the included and the excluded patients.

Parameters	Included	Excluded	P value
Eyes (patients), n	13 (12)	10 (10)	-
Age	71.2 ± 10.7	78.4 ± 8.76	.097
Male patients, n (%)	6 (50.0)	5 (50.0)	1.00
Right eyes, n (%)	6 (46.2)	6 (60.0)	.407
Pre-operative BCVA	.500 ± .148	.312 ± .211	.024*
Signal strength score	6.85 ± .555	5.30 ± 1.25	.001*
LOCS-N, median (range)	4 (3–6)	4 (4–6)	.030*
LOCS-C, median (range)	3 (3–5)	4 (3–4)	.011*
LOCS-P, median (range) ^†^	4 (3–5)	4 (3–5)	.956

BCVA = best-corrected visual acuity; LOCS = Lens Opacities Classification system; N = nuclear; C = cortical; P = posterior subcapsular.

Numeric data are presented as means ± standard deviations where applicable.

*P < .05.

^†^ LOCS-P scales were recorded in 4 included eyes and 5 excluded eyes.

The follow-up analysis was thus conducted in the 13 included eyes. Mean interval of the postoperative follow-up examination was 7.4 ± 4.6 days. The mean laser flare photometry value after surgery was 23.4 ± 21.2 pu/ms (range in healthy eyes: 0–10 pu/ms). No pseudophacic cystoid macular edema was observed after surgery. Both mean BCVA and mean signal strength score significantly improved after the surgery (Table 2).

**Table 2:** Comparison of pre- and post-operative characteristics in the included eyes.

Parameters	Pre-operative	Post-operative	P value
BCVA	.500 ± .148	.800 ± .230	.004*
Signal strength score	6.85 ± .555	7.54 ± .660	.013*

BCVA = best-corrected visual acuity.

Numeric data are presented as means ± standard deviations where applicable.

*P < .05.

### Microvasculature parameters

All parameters of perfusion density and vessel density significantly increased after the cataract surgery, except the average 3x3mm SCP vessel density with a p-value slightly above .05. The preoperative SCP perfusion density in the inner ring and inner 3.0 mm was .391 ± .032 and .374 ± .031, respectively, which significantly increased to .424 ± .020 and .405 ± .018 after the surgery (P = .04 and P = .03, respectively); the preoperative DCP perfusion density of the inner ring and inner 3.0 mm was .197 ± .060 and .176 ± .052, respectively, which significantly increased to .267 ± .061 and .239 ± .055 after the surgery (both, P = .001). The preoperative SCP vessel density in the inner ring and inner 3.0 mm was 18.9 ± 1.49 and 18.0 ± 1.37 U/mm, respectively, which significantly increased to 20.1 ± 1.75 and 19.2 ± 1.66 U/mm after the surgery (P = .047 and P = .035, respectively); the preoperative DCP vessel density in the inner ring and inner 3.0 mm was 9.55 ± 3.32 and 8.53 ± 2.87 U/mm, respectively, which significantly increased to 12.7 ± 2.74 and 11.3 ± 2.44 U/mm after the surgery (P = .04 and P = .03, respectively). Generally, the increase in DCP was more profound than that in SCP. (Table 3)

**Table 3:** Comparison of pre- and post-operative microvasculature parameters.

Layer	Location	Pre-operative	Post-operative	P value
**Perfusion density (unitless)**				
SCP	Inner ring	.391 ± .032	.424 ± .020	.004*
	Inner 3mm	.374 ± .031	.405 ± .018	.003*
	3 × 3 mm	.380 ± .031	.410 ± .021	.004*
DCP	Inner ring	.197 ± .060	.267 ± .061	.001*
	Inner 3mm	.176 ± .052	.239 ± .055	.001*
	3 × 3 mm	.187 ± .051	.250 ± .051	.002*
**Vessel density (inverse mm)**				
SCP	Inner ring	18.9 ± 1.49	20.1 ± 1.75	.047*
	Inner 3mm	18.0 ± 1.37	19.2 ± 1.66	.035*
	3 × 3 mm	18.1 ± 1.32	19.2 ± 1.67	.053
DCP	Inner ring	9.55 ± 3.32	12.7 ± 2.74	.004*
	Inner 3mm	8.53 ± 2.87	11.3 ± 2.44	.003*
	3 × 3 mm	9.12 ± 2.92	12.0 ± 2.59	.008*

SCP = superficial capillary plexus; DCP = deep capillary plexus; ETDRS = Early Treatment Diabetic Retinopathy Study

Data are presented as means ± standard deviations. Perfusion and vessel density were analyzed in the inner 3 mm ETDRS ring (inner ring), the central 1 mm plus the 3 mm ETDRS ring (inner 3 mm) and the whole 3 mm × 3 mm scan area (3 × 3 mm).

*P < .05.

Laser flare value was negatively correlated with all the density parameters in both layers. The correlation was comparatively stronger in the SCP. The Spearman’s correlation coefficients (rho) with perfusion and vessel density in the SCP were respectively -.454 (P = .030) and -.334 (P = .119). No correlation was found between laser flare value and FAZ parameters (rho: -.208, -.186, -.172, respectively for FAZ area, perimeter and circularity index).

Preoperative FAZ assessments were not available in 2 eyes because of the image quality, thus inferential analysis was carried out in the other 11 eyes. No significant changes of any FAZ parameters were noticed after the cataract surgery. (Table 4)

**Table 4:** Fovea avascular zone measurements on SCP.

Parameter	Pre-operative	Post-operative	P value
Area (mm^2^)	.231 ± .082	.215 ± .097	.441
Perimeter (mm)	2.31 ± .781	1.94 ± .443	.061
Circularity index	.589 ± .168	.678 ± .086	.171

Data are presented as means ± standard deviations. FAZ measurements were not available in 2 eyes, thus the analysis was carried out in 11 eyes.

### Image grading

**Fig 1:**
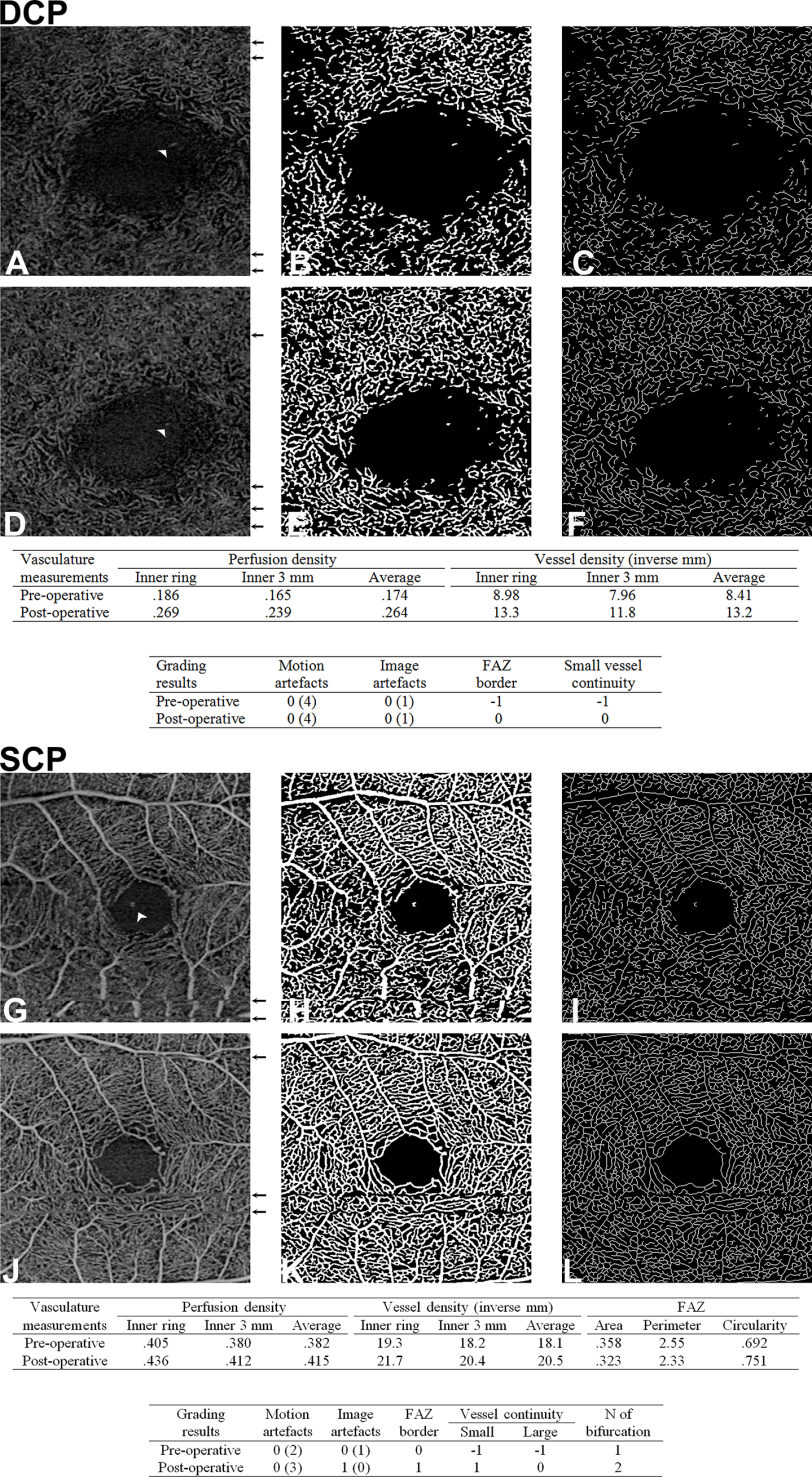
Representative example of pre and post cataract surgery outlining the improved vessel continuity and visibility and the reduction in artefacts after cataract surgery. A 79-year-old male cataract patient. In the DCP (A-F), 4 motion artefacts (black arrow) and one projection artefact (white arrow head) were found prior surgery (A). Perfusion density (B) and vessel density (C) were quantitatively measured. After surgery, same amount of artefacts were detected in the en-face image (D). Perfusion density (E) and vessel density (F) apparently increased. Scores of FAZ distinguishability and vessel continuity improved as well. In the SCP (G-L), 2 motion artefacts and one projection artefact were noticed before surgery (G). Severe motion artefacts disrupting the vessel continuity of the larger vessels. Perfusion density (H) and vessel density (I) were higher than in the DCP. After the surgery, 3 mild motion artefacts and no projection artefact were noticed (J). Perfusion density (K) and vessel density (L) apparently increased. FAZ was very well-defined. Scoring results (number of artefacts) are presented in the Table 6.

Cohen's kappa value for inter-reader agreement of independent image grading results varied between .31 and .63. (Table 5) After the consensus grading dataset was determined, significantly better vessel continuity and distinguishability of FAZ border were observed in the postoperative en-face slabs of both SCP and DCP. In the SCP images, the final normalized score of distinguishability of FAZ border, continuity of small and large vessels significantly increased from -23 to 77 (P = < .001), from -38 to 33 (P = .028) and from -46 to 62 (P = .003), respectively. In the DCP images, the final score of distinguishability of FAZ border and continuity of small vessels significantly increased from -62 to -8 (P = .026) and from -77 to 23 (P = .003), respectively. As a parameter of vessel visibility, number of discernable bifurcations in the SCP slab images presented a rising trend from .923 to 1.62 after surgery but did not show statistical significance. Similarly, strong decreases in motion and image artifacts were noticed in both SCP and DCP images, but these changes were not statistically significant: Mean number of motion artifacts in SCP and DCP numerically decreased by 37% (P = .089) and 42% (P = .080), respectively. The final normalized score of image artifacts of both layers numerically improved from 23 to 85 (P = .077). Most image artifacts in SCP and DCP were related to projections and signal loss. (Table 6) A representative example of “increased vessel continuity and visibility with less artifacts” can be found in Fig 1.

**Table 5:** Cohen's kappa value for inter-reader agreement of independent image grading.

Parameters	Kappa value	P value
Motion artefacts	.583	< .001*
Image artefacts	.313	.006*
Distinguishability of FAZ	.628	< .001*
Continuity of small vessels	.414	.002*
Continuity of large vessels	.513	< .001*

FAZ = fovea avascular zone.

*P < .05.

**Table 6:** Quantitative and qualitative evaluation of vessel continuity, vessel visibility and presence of artefacts in the SS-OCTA images.

Layer	Parameters	Pre-operative	Post-operative	P value
SCP	Motion artefacts	-54	8	.061
	N of motion artefacts	4.77 ± 2.09	3.00 ± 2.94	.089
	Image artefacts	23	85	.077
	Distinguishability of FAZ	-23	77	< .001*
	Continuity of small vessels	-38	33	.028*
	Continuity of large vessels ^†^	-46	62	.003*
	N of bifurcations ^†^	.923 ± .760	1.62 ± .290	.082
DCP	Motion artefacts	-31	23	.133
	N of motion artefacts	3.15 ± 1.86	1.84 ± 1.91	.080
	Image artefacts	23	85	.077
	Distinguishability of FAZ	-62	-8	.026*
	Continuity of small vessels	-77	23	.003*

FAZ = fovea avascular zone; SCP = superficial capillary plexus; DCP = deep capillary plexus; N = number.

The consensus grading contained an individual score for each feature of each image. Individual scores ranged from -1/0/+1, and were then summed up and normalized to a final score as presented in the Table. The maximal and minimal possible final scores ranged from -100 to +100. P-values were calculated by Pearson’s χ^2^ test. Number of motion artifacts and bifurcations were also analyzed and presented as means ± standard deviations in the Table. P-values were calculated by Student’s t-test.

*P < .05.

^†^ Continuity of large vessels and number of bifurcations were only graded in SCP images.

## Discussion

Many studies showed that OCTA can be used to ocular pathology monitoring as it is easy to use, has short acquisition times and is noninvasive. However, it is still limited in detecting certain anatomical features, such as microaneurysms, vessel loops, leakage, and some vessel segments.[10,16] This prospective study is the first to illustrate that cataract can significantly influence quantitative vasculature measurements even in high quality images using SS-OCTA.

In this study, a macular density algorithm was employed to analyze the microvasculature on OCTA images. Two parameters, perfusion density and vessel density, were measured in different ways to illustrate the microvascular anatomy. OCTA is knowns to be a reliable tool to detect these parameters with a high degree of repeatability.[14,17–19] To analyze the perfusion density, the total area of perfused vasculature per unit is evaluated. Larger vessels influence the measurement more than smaller capillaries. In contrast, the parameter vessel density treats all vessels equally: The retinal vasculature is untangled, its length is measured and divided by the area it originally occupied. The vessel density has a higher sensitivity to the loss of individual capillaries, but it also more prone to noise.

In our study, capillary density was greater in SCP than in DCP. This is in accordance to a previous study using a Zeiss instrument.[20] Besides, both perfusion and vessel density significantly increased after the surgery, while number and severity of artefacts did not significantly change. Meanwhile, among the quality parameters, distinguishability of the FAZ border and continuity of the vessels both significantly improved; the number of identifiable bifurcations increased, although the change was not significant. Of course, one may argue that the increase in vascular density is “real” due to an increased inflammatory status after surgery, leading to increased perfusion. However, the sophisticated autoregulation of retinal blood flow is believed to sufficiently compensate for variation in perfusion pressure, therefore to stabilize the retinal perfusion.[21,22] Increased oxygen saturation and accelerated blood flow velocity are believed to compensate for the potentially increased oxygen consumption after intraocular surgery, rather than enlarged retinal vessel caliber.[23] This compensation has been observed not only in retinal main vessels but also in perifoveal capillaries.[24–26] Although all these measurements were not acquired immediately after surgery but rather later after the intraocular intervention, they seem to support our observation that the caliber of retinal vessel remain stable after cataract surgery. Additionally, as a quantitative measurement of inflammatory response, flare was found to be negatively correlated with density parameters. This result not only suggests that flare (like cataracts) impairs the density measurements, but also strengthens the assumption that the increased density parameters are induced by improved visibility. Considering the numerical decrease of artefacts, the significant improvement of the vessel continuity (which can be also seen in Fig 1), the increase in the signal strength and the significant change in the quantitative parameters, we believe that the lower density values prior surgery are associated with the lower image quality due to media opacities.

In a recent study, Told et al. compared the influence of cataract between SD- and SS-OCTA images in 6 eyes with active nAMD. Cataract was found not to influence OCTA image quality and no significant difference between the two devices was found.[27] In this respective study, however we have to consider that the LOCS scale of the included patients with cataract was much lower (median LOCS scale was N2, C1, P2) and patients did not present with a significant, vision interfering cataract requiring surgery. Thus, given that they emphasized on coexisting cataract cases in nAMD patients, the good performance of SD- and SS-OCTA might be related to the lower cataract severity. In contrast, our study focused on patients with clinically significant cataract (LOCS scale: median: N4, C3, P4) without any concomitant retinopathies. Based on our findings it seems likely that the presence of a clinical significant cataract will result in an impaired evaluation of the microvasculature in patients with concomitant retinopathies or CNV. Notably, the analysis was only conducted in 13 eyes with adequate signal strength. 10 patients with low quality scan due to more severe cataract had to be excluded at the recruiting stage. Cataract severity and corresponding OCTA signal decrease were significantly stronger in the excluded patients. Based on this finding it seems very likely that OCTA devices may overcome early stage cataracts but may fail in case of clinical significant cataract.

With a longer wavelength of approximately 1060 nm, SS-OCT devices provide better tissue penetration, as well as better detection of signals from the deeper layers. This allows SS-OCT system to be less influenced by media opacity compared to normal SD-OCT.[4] Despite this favorable property, quantitative vessel parameters differed before and after cataract surgery with SS-OCTA. That implies that clinical significant lens opacities have an influence on SS-OCTA image assessment and should be taken into account when analyzing patients with cataract. It seems likely that SD-OCTA assessment may be even more impaired, but this assumption needs further investigation.

Interestingly, in contrary to the significant changes of vessel and perfusion density, FAZ measurements were found to be quite robust, despite the fact that the distinguishability of FAZ border was significantly better after the surgery at least from the graders’ perspective. FAZ dimension reflects the condition of the capillary circulation in the fovea area and has a strong positive correlation with the severity of capillary drop-out in several retinal vascular diseases.[28] FAZ area, perimeter and circularity index are the most common parameters. OCTA is capable of producing highly reproducible FAZ images.[8,28,29] and has been shown to reliably identify the changes of the perifoveal capillary circulation.[30] Since the perifoveal capillary circulation seems only differed in terms of oxygen saturation and blood flow velocity,[25,26] the solid results of the FAZ measurements are believed to be reliable and robust. It has to be noted that the algorithm was not able to perform preoperative FAZ measurements in two eyes with lower image quality, which highlights the limitation of automated FAZ measurements with coexisting media opacities. This limitation however may be of advantage, because it prevents wrong assessment and increases the reliability of the FAZ measurements.

In previous studies, media opacities were confirmed to be a reason for signal loss.[5] And Al-Sheikh et al. found that in healthy volunteers, lower image quality was associated with artefact frequency and measurement repeatability.[31] We noticed a numeric decline in mean number of motion artifacts in both SCP (by 37%) and DCP (by 42%) after cataract surgery. Although all images were taken under tracking mode, such artefacts seem unavoidable with the current imaging technique. Real-time eye motion tracking technology has been shown to provide high-fidelity and motion-free OCTA images in case of relatively small eye movements and overcome unstable fixation. Nevertheless, larger eye movements due to fixation loss in patients with clinical significant cataract may still impair the acquisition of volumetric data and lead to motion artefacts.[32]

Image artifacts were generally not severe in our study and did not significantly change after cataract surgery. Some common image artifacts, such as projection- and segmentation errors can be partially corrected using proprietary software or manual adjustment.[7] Despite the utilization of proprietary software, a few projection artifacts in the DCP images we still observed pre as well as post cataract surgery. Similar findings were also reported in previous studies, [7,12] which indicates that respective software needs continuous improvements.[33]

One limitation of our study may be that only patients with clinical significant, vision deteriorating cataract were included in this analysis. The impact of early stage cataract was not evaluated. Secondly, we did not measure the laser flare value before the cataract surgery, as we intended to quantify the post-operative inflammatory response. A more entire analysis could have been acquired if the pre-operative flare value were available.

In conclusion, we investigated the accuracy and reliability of SS-OCTA application in visually relevant cataracts. A significant influence of cataract on both image quality and retinal blood flow measurements was confirmed. Also inflammation quantified using laser flare photometry was negatively correlated with the density parameters, indicating that media opacity of any kind impact quantitative assessment of density parameters. FAZ measurements seemed to be the most reliable variables. As the majority of patients with retinal pathologies involving the retinal or choroidal vasculature are of older age or may show earlier development of cataract due to their underlying disease, the impact of cataract on the OCTA assessment should always be taken into consideration. Images should be carefully checked for discontinuous vessels, also in high quality images and absence of artefacts. Ophthalmologists are encouraged to take note of the lens condition before accessing quantitative OCTA data and retinal vasculature using SS-OCTA.
